# Women's satisfaction with comprehensive abortion care services and associated factors in central Gondar zone public primary hospitals, northwest Ethiopia, 2023

**DOI:** 10.3389/frph.2024.1400359

**Published:** 2024-10-01

**Authors:** Nebiyu Solomon Tibebu, Melaku Birhanu Alemu, Bayew Kelkay Rade, Belayneh Ayanaw Kassie, Mequanint Melesse Bicha, Muhabaw Shumye Mihret, Getachew Muluye Gedef

**Affiliations:** ^1^ Department of Clinical Midwifery, School of Midwifery, College of Medicine and Health Sciences, University of Gondar, Gondar, Ethiopia; ^2^ Department of Health Systems and Policy, College of Medicine and Health Sciences, University of Gondar, Gondar, Ethiopia; ^3^ Department of General Midwifery, School of Midwifery, College of Medicine and Health Sciences, University of Gondar, Gondar, Ethiopia; ^4^ Department of Women’s and Family Health, School of Midwifery, College of Medicine and Health Sciences, University of Gondar, Gondar, Ethiopia; ^5^ Department of Obstetrics and Gynecology, College of Medicine and Health Sciences, University of Gondar, Gondar, Ethiopia

**Keywords:** abortion care service, Women's satisfaction, sexual and reproductive health, public hospital, Ethiopia

## Abstract

**Background:**

Abortion complications are the leading causes of maternal death in low and middle-income countries, including Ethiopia. Providing quality and comprehensive abortion care services is crucial for improving the health of women and increased their satisfaction. Evaluating a client's satisfaction with abortion care is clinically relevant since women's satisfaction with health services is one of the key indicators of high-quality healthcare services. Therefore, this study aimed to assess women's satisfaction with comprehensive abortion care services and associated factors.

**Methods:**

An institution-based cross-sectional study was implemented among 333 women in Central Gondar Zone public primary hospitals from October 1, 2022, to April 30, 2023. Eligible participants were selected using a systematic random sampling technique. The data was collected using an interviewer-administered semi-structured, and pretested questionnaire. STATA version 17 and SPSS version 25 software were used for data entry and analysis respectively. Bivariable and multivariable logistic regression models were used to identify factors associated with clients’ satisfaction with comprehensive abortion care services. A *P*-value of ≤0.05 with a 95% confidence interval was the cutoff point for determining statistical significance.

**Results:**

This study revealed that the level of client satisfaction with comprehensive abortion care services was 60.4% (95% CI: 55.0%, 66.0%). The use of abortion medication (AOR = 4.41, 95% CI: 2.59, 7.48), women's age 20–24 years (AOR = 2.94, 95% CI: 1.02, 8.48), and being a student (AOR = 2.88, 95% CI: 1.10, 7.51) were significantly associated with women's satisfaction with comprehensive abortion care services.

**Conclusions:**

Women's satisfaction with comprehensive abortion care services was relatively low, and it was strongly correlated with the method of abortion, age, and occupation. To improve women's satisfaction requires a comprehensive understanding of women's values and perspectives, providing sexual and reproductive health education, and quality abortion care services are recommended.

## Introduction

Abortion is defined by the World Health Organization (WHO) as pregnancy termination prior to fetal viability; however, several nations use different gestational age and/or fetal weight to determine the viability of the fetus (1). Depending on the cause, an abortion can be spontaneous or induced, and the procedure can be carried out using medication or through surgical intervention (2, 3). To further differentiate between abortions, the words “therapeutic abortion” and “voluntary abortion” are employed; therapeutic abortion refers to an abortion performed to save the life of the pregnant woman in cases where continuing the pregnancy poses a serious risk to the mother's life, while voluntary abortion is chosen for personal or social reasons (4).

Ethiopia's 2005 Penal Code and the Ministry of Health's guidance on safe abortion services allow abortions under specific circumstances, such as if the pregnancy is a result of rape or incest; if the continuation of pregnancy endangers the life of the woman, if the fetus has a serious and incurable deformity; or if the pregnant woman is physically and mentally unfit to raise the child due to a physical or mental disability or her minority. Despite this, unsafe abortions remain prevalent in Ethiopia, contributing significantly to maternal morbidity and mortality (5, 6).

Abortion care is a crucial aspect of reproductive health services, and ensuring high-quality and accessible abortion care services is essential for promoting women's health, rights, and satisfaction (7). The WHO has made it very clear in recent years that access to high-quality healthcare, including services related to abortion, is a prerequisite for protecting the right to health as a fundamental human right (8).

Worldwide, nearly 22 million unsafe abortions are carried out annually, and the majority of them occur in low- and middle-income countries (9). Approximately 13% of maternal deaths in developing nations are attributable to unsafe abortion practices (10, 11). In Ethiopia, complications from abortions are the leading cause of maternal morbidity and mortality; about 31% of all maternal mortalities are due to unsafe abortions (12).

Strict national laws, a lack of readily available safe abortion services, and inadequate care following an abortion have caused many women to die too young (13). Maternal death and morbidity associated with abortion are clearly avoidable via comprehensive sexual education, easily accessible family planning services, and safe and legal-induced abortion (14).

Comprehensive abortion care (CAC) is a successful and holistic approach for preventing unintended pregnancy and repeated abortion, managing abortion complications, and eventually increasing client satisfaction (15).

Effective and quality services are essential for improving women's health outcomes and, at the same time, satisfying the healthcare needs and rights of women who have experienced abortion complications (16).

Client service-related satisfaction levels are important for evaluating the quality of healthcare offered by the institution, identifying what factors affect clinical performance and patient retention, and ascertaining whether the services offered meet the needs of the clients, such as having a choice of services, positive interactions with providers, receiving accurate and complete information, and receiving high-quality care (17).

Client satisfaction with abortion services is a relevant and widely used indication of the quality of healthcare provided; it influences women's desire to seek abortion treatment and return for post abortion care and eventually significantly impacts health outcomes (15, 18). Satisfied clients are more likely to adhere to treatment plans, participate actively in their care, continue utilizing services, and recommend others to services. Conversely, low-quality care services may lead women to seek care from unqualified providers or to self-induce abortions, which increases the risk of morbidity and death from abortion (19).

Access to quality abortion care services remains a challenge in low-income nations (17). Continuous service improvement initiatives are necessary to maintain service quality and meet women's healthcare needs. Ethiopia has made progress in providing more access to reproductive healthcare services, including abortion, but there are still gaps in policy objectives and actual implementation, especially in poor and rural areas. Comprehending women's contentment with abortion care services is crucial not only for assessing the quality of services but also shaping policy and practice to better meet women's needs. Therefore, it is reasonable to evaluate client satisfaction with CAC services to identify areas that need attention to improve service quality and obtain better results.

Women's satisfaction with abortion services is influenced by various factors such as the condition of medical facilities and equipment (cleanliness and safety protocols), the skill level and expertise of the healthcare providers; counseling and information provision (clear and comprehensive information about the procedure, risks, and options before the procedure and obtaining informed consent); personal factors (women's prior experiences with healthcare services, level of education and awareness about abortion and reproductive health); cultural and societal factors (cultural sensitivity and personal beliefs towards abortion, support from family and community members); emotional and psychological support, legal environment (restrictions or protections regarding abortion services); timeliness (length of time women have to wait for appointments and procedures) and privacy and confidentiality during the care process (availability of private spaces for consultations and procedures) (7, 13, 15, 16, 20–26).

Previous studies in Ethiopia have primarily focused on the magnitude of abortion services, but little attention has been given to evaluating women's satisfaction with CAC. Therefore this study aims to assess satisfaction levels and to identify associated factors, and to provide actionable insights for healthcare providers and policymakers.

## Materials and methods

### Study design, setting and period

An institution-based cross-sectional study was carried out in the Central Gondar zone, Amhara Regional State, Ethiopia, from October 1, 2022 to April 30, 2023. The Central Gondar zone is located 748 km from Addis Ababa, Ethiopia, and 182 km from Bahir Dar, the capital town of the Amhara Regional State. It covers thirteen woreda with an estimated total population of 2,896,928. In this zone, there were 14 districts, 9 hospitals and 75 health centers.

### Study populations

All women who utilized abortion care in public primary hospitals in the central Gondar Zone during the study period.

### Sample size and sampling procedures

Using a single population proportion formula and the following presumptions; prevalence (P) of client satisfaction with abortion care services: 26.9% (27), with a 95% confidence interval (CI) and 5% margin of error; the sample size was calculated as follows:

$n=\frac{Z\alpha /2{)}^{2}p(1-p)}{d^{2}}\;=\frac{{\left(1.96\right)}^{2}\;0.27(1-\;0.27)}{{\left(0.05\right)}^{2}}=303$; by considering a 10% nonresponse rate, a final sample size of 333 was obtained.

where *n* is the required sample size, z is the standard normal distribution curve at 95% confidence level *α* = level of significance, p is the proportion of client satisfaction with abortion care services, and d is the margin of error.

Based on the total number of abortions conducted in the preceding month, a proportionate amount of the sample was assigned to each institution. The desired sample sizes from each hospital were then chosen through the application of a systematic random sampling technique.

### Data collection procedure

Data were collected using an interviewer-administered, semi-structured questionnaire. The data collection tool was prepared after relevant and related literature was reviewed. Ten midwives collected the data, and five master's-holder midwives supervised the data collection process. Moreover, the principal investigator was available to offer guidance when needed.

### Data quality control

The questionnaire was first written in English, then translated into Amharic, the local language, and then back into English. A pretest was carried out on five percent of the sample size before the actual data collection to verify the tool's applicability and linguistic clarity. Half a day training was provided for the data collectors and supervisors to help them better understand the purpose of the study and the overall procedure of the data collection process. Supervisors and the principal investigator conducted daily checks on the questionnaire to ensure its completeness during the actual data collection period.

### Data management and analysis

After the data were checked for accuracy, consistency, and missing values, STATA version 17 and SPSS version 25 software were used for data entry and analysis respectively.

Descriptive statistics were computed to characterize the study population in terms of sociodemographic and other pertinent factors. The relationship between each independent variable and outcome variable was initially determined by bivariable logistic regression analysis and variables with a *p*-value less than 0.2 were subsequently included in the multivariable analysis. Finally, variables with a *p*-value ≤ 0.05 were considered to be significantly associated factors with women's satisfaction with CAC services. The direction and strength of the associations were determined by the adjusted odds ratio (AOR). Hosmer-Lemeshow goodness of fit test was used to evaluate the fitness of the final model.

### Operational definition

**Comprehensive abortion care** refers to providing safe induced abortion for all legal indications allowed by national law as well as providing elements of post abortion contraceptives (28).

#### Measurements

Women's satisfaction with CAC was measured using a composite variable with multiple indicators (physical environment, technical quality of provider, information provision, privacy and confidentiality). The satisfaction level of women was measured by 20 five-point Likert scale questions ranging between 0 and 4 (0 = neutral, 1 = strongly disagree, 2 = disagree, 3 = agree & 4 = strongly agree). The scores for each domain were calculated by summing the answers to all items in each domain. The overall and component wise satisfaction scores were categorized into two groups based on the mean score. Women who scored mean and above were categorized as satisfied, whereas those who scored below the mean were categorized as unsatisfied (13, 15, 29).

### Ethics approval and informed consent

The study followed the principles outlined in the Declaration of Helsinki. Ethical approval was obtained from the Ethical Review Board (30) of the University of Gondar (Reference number: 189/11/2022). A permission letter for each hospital was obtained from the Amhara Public Health Institute. Before data collection, the study participants were informed about the purpose of the study and their right to decline participation or discontinue the interview at any time. Written informed consent was obtained from each participant and/or their caregivers or legal representatives for underage and illiterate participants on their behalf. Their confidentiality was maintained by omitting personal identifiers from the data collection tool.

## Results

### Sociodemographic characteristics of the study participants

A total of 333 women were interviewed, yielding a 100% response rate. The mean age of the study participants was 25.3 years (standard deviation ± 4.9). Of the total participants, the majority (82.6%) were Orthodox Christians by religion, 58% lived in urban areas, and 46.5% had no formal education. (Table 1).

**Table 1:** Sociodemographic characteristics of the study participants in public primary hospitals of central Gondar zone, Amhara regional state, Ethiopia, 2022/2023 (*n* = 333).

Variables	Category	Frequency	Percent
Maternal age	14–19	46	13.8
	20–24	73	21.9
	25–34	154	46.2
	≥35	67	18.3
Residence	Rural	140	42.0
	Urban	193	58.0
Marital status	Married	204	61.3
	Single	122	36.6
	Others^a^	7	2.1
Religion	Orthodox	275	82.6
	Muslim	58	17.4
Education	No formal education	155	46.5
	Elementary education	36	10.8
	Secondary and above	57	17.1
	College and above	85	25.5
Occupation	Housework	120	36.0
	Employee	95	28.5
	Private business	45	15.7
	Student	58	17.4
	Others^b^	15	4.5
Sex preference for your CAC services	Male	150	45.0
	Female	183	55.0
Do you earn/make money by yourself?	Yes	182	54.7
	No	151	45.3

^a^ Divorced, widowed.

^b^ Daily laborer, housemaid.

### Abortion care-related characteristics of the study participants

More than half 184(55.3%) of the participants were experienced first trimester abortion by gestational age (GA), and 177(53.2%) of the participants utilized abortion medication as a method of uterine evacuation (Table 2).

**Table 2:** Abortion care-related characteristics of the study participants in public primary hospitals of central Gondar zone, Amhara regional state, Ethiopia, 2022/2023 (*n* = 333).

Variables	Categories	Frequency	Percentage
Gestational age	First trimester	184	55.3
	Second trimester	126	37.8
	Unknown	23	6.9
Diagnosis type	Induced abortion	199	59.8
	Postabortion care	134	40.2
Procedure type	Medical abortion	177	53.2
	Manual vacuum aspiration	156	46.8
Informed about the availability of post-abortion services	Yes	184	53.3
	No	149	44.7
Any post abortion family planning method used	Yes	101	30.3
	No	232	69.7

### Level of women's satisfaction with the given abortion care services

**Figure 1:**
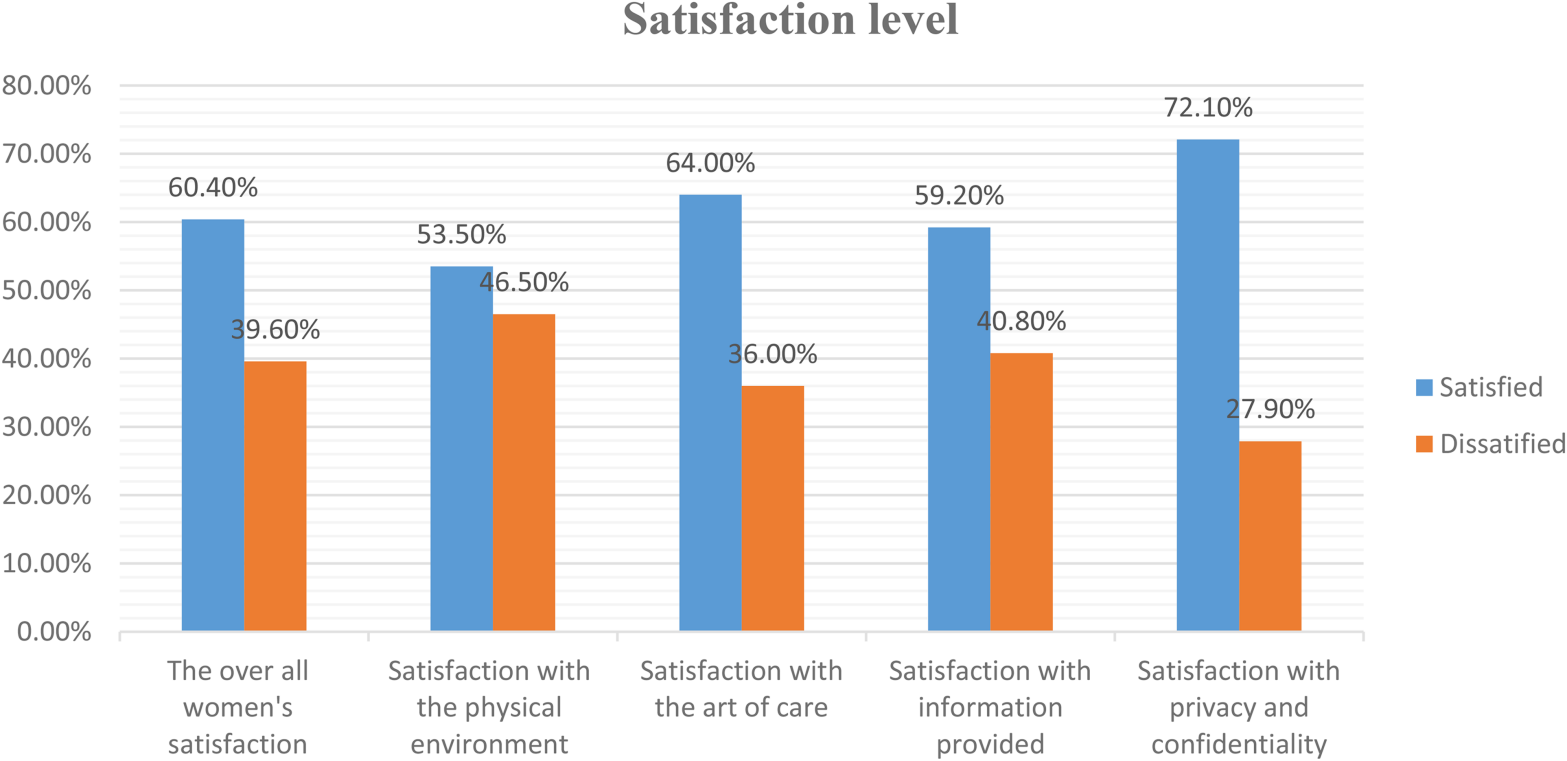
Level of women's satisfaction with each item dimension and overall satisfaction with comprehensive abortion healthcare services in central Gondar zone public primary hospitals, Amhara regional state, Ethiopia, 2022/23.

Participants were asked to assess their level of satisfaction with the given abortion healthcare services using five item dimensions, such as the physical environment, quality of care, information provision, and privacy and confidentiality. In this analysis, women's satisfaction with privacy and confidentiality accounted for the highest proportion (72.1%). Overall women satisfaction with comprehensive abortion care services was 60.4%, with a 95% CI (55.0, 66.0) (Figure 1).

### Factors associated with the level women's satisfaction

To identify the variables related to women's satisfaction, bivariable and multivariable logistic regression analysis were performed. According to the bivariable logistic regression analysis, women's age of 20–24 and 25–34 years, being a rural resident, being a student, and utilizing abortion medication were associated with satisfaction. In the final model women's age of 20–24 years, occupation as a student and use of abortion medication were independently and significantly associated with women's satisfaction.

Compared to women under twenty years of age, women aged twenty to twenty-four years had 2.94 times greater odds of being satisfied with their CAC services (AOR = 2.94, 95% CI: 1.02, 8.48). Similarly, the odds of women's satisfaction with CAC services among women whose occupation was student were 2.88 times higher than those among whose occupation was housework (AOR = 2.88; 95% CI: 1.10, 7.51). Furthermore, respondents who utilized abortion medication as a method of uterine evacuation were 4.41 times more likely to be satisfied with CAC services than that of those who used the surgical method (AOR = 4.41, 95% CI: 2.59, 7.48) (Table 3).

**Table 3:** Bivariable and multivariable analysis results to identify associated factors with women's satisfaction with CAC services in central Gondar zone public primary hospitals, Amhara regional state, Ethiopia, 2022/2023.

Variables	Category	Level of satisfaction		COR 95%CI	AOR 95%CI	*P*-value
		Satisfied	Unsatisfied			
Maternal age	14–19	30 (14.9)	16 (12.1)	1	1	
	20–24	58 (28.9)	15 (11.4)	**2.06** (**0.89–4.73)**	**2.94** (**1.02–8.48)**	0.047
	25–34	78 (38.8)	76 (57.6)	**0.44** (**0.33–1.65)**	0.82 (0.31–2.17)	0.687
	≥35	35 (17.4)	25 (18.9)	0.75 (0.43–1.99)	1.26 (0.43–3.69)	0.672
Residence	Rural	78 (38.8)	62 (47.0)	0.72 (0.46–0.12)	0.77 (0.37–1.62)	0.492
	Urban	123 (61.2)	70 (53.0)	1	1	
Marital status	Married	120 (59.7)	84 (63.6)	1	1	
	Single	75 (37.3)	47 (35.6)	1.12 (0.71–1.77)	1.34 (0.77–2.35)	0.305
	Others^a^	1 (0.8)	6 (3.0)	**4.20** (**0.49–35.53)**	6.43 (0.63–65.49)	0.116
Religion	Orthodox	162 (85.6)	113 (80.6)	0.69 (0.38- 1.27)	0.76 (0.36–1.69)	0.521
	Muslim	39 (19.4)	19 (14.4)	1	1	
Education	No formal education	83 (41.3)	72 (54.5)	**0.63** (**0.36–1.08)**	0.53 (0.20–142)	0.208
	Elementary school	25 (12.4)	11 (8.3)	1.24 (0.53–2.86)	1.15 (0.31–4.04)	0.826
	Secondary school	38 (18.9)	19 (14.4)	1.09 (0.54–2.21)	0.53 (0.19–1.47)	0.223
	College and above	55 (27.4)	30 (22.7)	1	1	
Occupation	Housework	67 (33.3)	53 (40.2)	1	1	
	Employee	56 (27.9)	39 (29.5)	1.09 (0.66–1.96)	1.36 (0.63–2.91)	0.429
	Private business	25 (12.4)	20 (15.2)	0.98 (0.49–1.97)	1.13 (0.45–2.86)	0.800
	Student	44 (21.9)	14 (10.6)	**2.49** (**1.23–5.25)**	**2.88** (**1.10–7.51)**	0.030
	Others^b^	9 (4.5)	6 (4.5)	1.19 (0.39–3.54)	1.24 (0.33–4.65)	0.750
Sex preference for CAC services	Male	97 (48.3)	53 (40.2)	1	1	
	Female	104 (51.7)	79 (59.8)	0.72 (0.46–1.12)	0.73 (0.41–1.29)	0.281
Do you earn money by yourself?	Yes	110 (54.7)	72 (54.5)	1.00 (0.64–1.57)	0.99 (0.54–1.81)	0.973
	No	91 (45.3)	60 (45.5)	1	1	
GA in Weeks	1st TM	131 (65.2)	53 (40.2)	**1.90** (**0.78–4.60)**	2.41 (0.89–6.59)	0.085
	2nd TM	57 (28.4)	69 (52.3)	0.63 (0.26–1.56)	0.70 (0.25–1.96)	0.505
	Unknown GA	13 (6.5)	10 (7.6)	1	1	
Diagnosis	Abortion induction	117 (58.2)	82 (62.1)	0.85 (0.54–1.33)	0.70 (0.41–1.19)	0.194
	Post abortion care	84 (41.8)	50 (37.9)	1	1	
Method	Medical	132 (65.7)	45 (34.1)	3.69 (2.32–5.88)	**4.41** (**2.59–7.48)**	0.001
	Surgical	69 (34.3)	87 (65.9)	1	1	
Post abortion family planning used	Yes	113(56.2)	71(53.8)	0.89 (0.55–1.43)	0.78(0.44–1.39)	0.408
	No	88(43.8)	61(46.2)	1	1	

GA, gestational age; TM, trimester; AOR, adjusted odds ratio; COR, crude odds ratio.

^a^ Divorced, widowed.

^b^ Daily laborer, no job.

* Bold letters: variables associated with the outcome variable in bivariable and multivariable analysis.

## Discussion

Abortion remains a complex and contentious issue globally, shaped by cultural, religious, legal, and healthcare factors. Several scientific studies have examined how satisfied patients are with their healthcare services, highlighting how crucial it is to make sure that the services are of a standard that meets patients’ expectations. Evaluating women's satisfaction with abortion care services in Ethiopia is especially important to make sure services meet women's needs, where access to complete reproductive health treatments is not satisfactory. It provides data for policy insight to improve the accessibility and quality of abortion services and patient-centered care. It also helps to create a healthcare system that upholds the reproductive rights of women, offers high-quality care, and advances their general wellbeing. Patients have specialized knowledge and experience that can be applied to enhance clinical outcomes, quality of care, patient experiences, patient autonomy, research relevance and impact, and public trust in research, it is crucial for them to participate collectively in research (30).

This study assessed the level of women's satisfaction and associated factors with comprehensive abortion care services in Central Gondar Zone public primary hospitals in the Amhara Regional State, Ethiopia. Thus, the prevalence of women's satisfaction with comprehensive abortion care services was 60.4%, with a 95% CI (55.0, 66.0). A woman's age between 20 and 24 years, being a student, and using abortion medication as a method of uterine evacuation were identified as factors associated with women's satisfaction.

The finding of this study are comparable to those of the studies done in Addis Ababa and Mojo town (15, 16). However, this result is lower than those of studies in Jimma (23), the Gambela region (13), and Kenya (31) and higher than those of studies done in northwest Ethiopia (17) and the Tigray region (25).

The variation in the study setting and time, sample size, and sociocultural background of the study subjects could be the cause of the disparity in the level of satisfaction with the services. One explanation for the lower prevalence in the current study might be because some previous studies evaluated the satisfaction levels of women who had only medical abortions. The observed discrepancy may also be explained by variations in the attitudes and abilities of healthcare providers, difficulties in adhering to clinical protocols, and a lack of appropriate equipment at the health institution.

Those respondents who utilized abortion medication for uterine evacuation were 4.41 times more likely to be satisfied with abortion care services than those who utilized surgical methods. This result is consistent with the research finding in Mojo town. One possible explanation is that women may experience pain and discomfort associated with the surgical process. Additionally, a lower level of satisfaction may also result from not receiving counseling or from not using pain management therapy during the procedure. Abortion patients should have access to accurate information and counseling both before and after the procedure. Providers should educate women on the characteristics of abortion methods during counseling sessions and assist them in choosing the best option for them (32).

However, this result contradicts the findings of Addis Abeba, which reported that women who underwent surgical abortion were more satisfied than women who received abortion medication (16). The possible reason might be the expectations and awareness of the respondents about the methods; women in urban residences have adequate information due to better access to various health facilities and more media exposure, which may increase their expectations that surgical methods are associated with pain and discomfort (16). Furthermore, in one randomized trial of women randomized to surgical abortion, 92% stated that they would choose a surgical method for their next abortion, whereas only 63% of women randomized to medical abortion would choose that option in the future (33).

The odds of women's satisfaction with comprehensive abortion care services were 2.94 times greater among women aged is between 20 and 24 years. This is might be because of young women may not have a better understanding of the risks and benefits of abortion in their lives, as they are focused only on ending unwanted pregnancies to avoid social stigma regardless of the standard of treatment.

Women's occupation is also a significant factor for women's satisfaction; being a student was highly likely to be more satisfied with comprehensive abortion care services compared to those whose occupations involved housework. This might be because students might have limited information about the quality of abortion services and low expectations anticipating that they may not be well treated by providers due to interference with their pregnancies, which means that less educated clients may not request for better services (15).

However, this finding differs from those of previous studies conducted in Jimma (23) and Addis Abeba (16), which reported that employed women were more likely to be satisfied with services than those in other occupations. Patients with greater education are able to express their needs and preferences more clearly, which enables medical professionals to give more individualized and fulfilling care. Patients feel more confident and satisfied with their healthcare experiences when they are equipped with the knowledge necessary to make educated decisions regarding their sexual health. Additionally, employed women have better communication with healthcare providers, which may help them understand the scarcity of resources available in health facilities to deliver procedures accordingly (17). Therefore, the possible reason for the discrepancy may be due to the difference in study settings and populations.

Comprehensive sexual and reproductive health (SRH) education plays a crucial role in increasing patient satisfaction and promoting overall health and well-being. It provides information on safe sex practices, contraception, helping to prevent sexually transmitted infections (STIs) and unwanted pregnancies, and enabling early detection of potential health issues. Therefore, healthcare providers can significantly enhance patient satisfaction and promote general health and well-being, which will result in healthier individuals and communities, by incorporating sexual health education into patient treatment.

In summary ensuring high-quality abortion care and improving women's satisfaction involves addressing multiple facets of service delivery, including clinical quality, emotional support, accessibility, and respect for women's autonomy. Comprehensive sexual and reproductive health education is also essential for empowering women to make informed decisions.

### The strengths and limitations of the study

The 100% response rate was maintained to investigate the multifactorial nature of women's satisfaction. Furthermore, this study identified contributing factors that grab attention to enhance the quality and increase women's satisfaction with CAC services. Some of the limitations of this study are that, although satisfaction is a process, it was assessed at one point in time, and this might underestimate the level of women's satisfaction. Given that the data were gathered through face-to-face interviews, social desirability bias might have been introduced.

## Conclusions

This study revealed that 60.4% of women were satisfied with comprehensive abortion care services. Women's age of 20–24 years, being a student, and utilizing a medical abortion were factors associated with women's satisfaction with CAC services. To improve women's satisfaction with abortion care services, requires a holistic approach to abortion care, including better information provision, enhanced privacy and confidentiality, and quality healthcare services. Thus, all the concerned bodies should focus on advancing these factors to improve the quality of care and increase women's satisfaction with CAC services. In order to fully understand factors that influence comprehensive abortion care services longitudinal study recommended.

## Data Availability

The Datasets are found from the corresponding author upon request.
